# Development of a physiologically based pharmacokinetic model of actinomycin D in children with cancer

**DOI:** 10.1111/bcp.12878

**Published:** 2016-02-25

**Authors:** Christopher Walsh, Jennifer J. Bonner, Trevor N. Johnson, Sibylle Neuhoff, Essam A. Ghazaly, John G. Gribben, Alan V. Boddy, Gareth J. Veal

**Affiliations:** ^1^ Northern Institute for Cancer ResearchNewcastle UniversityNewcastle upon TyneUK; ^2^ Simcyp Limited (a Certara Company)SheffieldUK; ^3^ Barts Cancer InstituteQueen Mary University of LondonLondonUK; ^4^ Faculty of PharmacyThe University of SydneyNSW2006Australia

**Keywords:** actinomycin D, cancer, paediatrics, pharmacokinetics, physiologically based pharmacokinetic modelling

## Abstract

**Aims:**

Use of the anti‐tumour antibiotic actinomycin D is associated with development of hepatotoxicity, particularly in young children. A paucity of actinomycin D pharmacokinetic data make it challenging to develop a sound rationale for defining dosing regimens in younger patients. The study aim was to develop a physiologically based pharmacokinetic (PBPK) model using a combination of data from the literature and generated from experimental analyses.

**Methods:**

Assays to determine actinomycin D Log P, blood:plasma partition ratio and ABCB1 kinetics were conducted. These data were combined with physiochemical properties sourced from the literature to generate a compound file for use within the modelling‐simulation software Simcyp (version 14 release 1). For simulation, information was taken from two datasets, one from 117 patients under the age of 21 and one from 20 patients aged 16–48.

**Results:**

The final model incorporated clinical renal and biliary clearance data and an additional systemic clearance value. The mean AUC_0‐26h_ of simulated subjects was within 1.25‐fold of the observed AUC_0‐26h_ (84 ng h ml^−1^ simulated *vs.* 93 ng h ml^−1^ observed). For the younger age ranges, AUC predictions were within two‐fold of observed values, with simulated data from six of the eight age/dose ranges falling within 15% of observed data. Simulated values for actinomycin D AUC_0‐26h_ and clearance in infants aged 0–12 months ranged from 104 to 115 ng h ml^−1^ and 3.5–3.8 l h^−1^, respectively.

**Conclusions:**

The model has potential utility for prediction of actinomycin D exposure in younger patients and may help guide future dosing. However, additional independent data from neonates and infants is needed for further validation. Physiological differences between paediatric cancer patients and healthy children also need to be further characterized and incorporated into PBPK models.

## What is Already Known about this Subject

- Treatment of children with cancer with actinomycin D is associated with hepatotoxicity, particularly in younger patients.
- Limited information currently exists relating to the pharmacokinetics of actinomycin D in either adult or childhood cancer patient populations.
- Current actinomycin D dosing guidelines for infants and younger children vary between clinical trial protocols.

## What this Study Adds

- We generated essential physicochemical property data for actinomycin D, facilitating the generation of a compound file for PBPK model development.
- A PBPK model for actinomycin D was developed, with the potential for prediction of actinomycin D exposure in infants and younger children, which may provide a useful tool to guide future dosing in these challenging patients.

## Introduction

Actinomycin D (Act D) is a polypeptide antibiotic that has been used in the treatment of various types of cancer for over 40 years. It functions by binding to DNA in a guanine‐dependent manner thus inhibiting RNA polymerase and preventing DNA transcription 1. Act D is predominantly administered to paediatric patients with Wilms' tumour, Ewing's sarcoma or rhabdomyosarcoma, often in combination with other chemotherapeutics including vincristine and cyclophosphamide. These three cancers account for roughly 10% of all cancers seen in children in the United States. While Ewing's sarcoma occurs predominantly in teens, both Wilms' tumour and rhabdomyosarcoma are prevalent in younger children, with Wilms' tumour affecting almost exclusively patients under 6 years of age. Treatment with Act D is associated with a type of hepatotoxicity known as sinusoidal obstructive syndrome (SOS) (formerly called veno‐occlusive disease) 2. SOS is caused by the blockage of the small blood vessels of the liver following chemotherapy or bone marrow transplant, with symptoms including fever, anaemia and thrombocytopenia. Age appears to be a risk factor for the development of SOS; with patients under three years of age exhibiting a 15% chance of developing the disease compared to a 4% likelihood in patients over three years of age 3. There is also evidence to suggest that dose intensity of act D may be linked to incidence of SOS, with one study showing that a dose reduction from 60 μg ml^−1^ to 40 μg ml^−1^ led to fewer toxic events 4.

Clinical investigations into the pharmacokinetics (PKs) of Act D have been limited, with a small published PK study (*n* = 3) in adult patients 5 and a more recent study of Act D, methotrexate and etoposide in combination (*n* = 35 adult patients) 6. The PK of Act D is typically described by a multicompartmental model, reflecting a rapid distribution phase into tissues and a long terminal phase half‐life 5. This long terminal phase reflects extensive distribution into many tissues, including skeletal muscle, kidney, lung and liver, with correspondingly long tissue half‐lives seen in animal studies 7. Low cerebrospinal fluid (CSF) concentrations after IV administration 5 as well as poor brain uptake in animal studies 7 indicate poor central nervous system (CNS) penetration. Act D does not bind strongly to red blood cells, but has been shown to concentrate in nucleated blood cells such as lymphocytes and granulocytes. Elimination is thought to be mainly via renal and biliary excretion, with approximately 35% of an administered dose eliminated in the urine and faeces after nine days. Metabolism is negligible, with 1–4% of an IV dose converted to monolactone metabolites 5.

There has been a greater interest in the PK of Act D in young patients, with larger sized published studies in children 8, 9, 10. These paediatric studies were carried out mainly due to concerns regarding the correlation between younger age and SOS in this population and the very limited amount of published data available from adult cancer patients. While population PK models have been developed for Act D 8, the associated studies enrolled very few patients under the age of one year and none who were preterm or immediately post‐natal. Given the risk factor of younger age in the development of SOS, it is important that consideration is given to how children under the age of three years are dosed with Act D, particularly in the case of neonates and infants. While malignancies in infants only represent approximately 10% of all cancers diagnosed in children under 15, Wilms' tumour has a relatively high incidence rate within the infant cancer patient population 11. Development of a rationale for dose selection in these very young patients should aim to maximize anticancer efficacy while minimizing the risk of toxicity.

No current consensus exists on the most appropriate dose and dosing regimen for Act D in younger children, with dose reductions implemented for children under 6 months, under 12 months or under 3 years, depending on geographic region (e.g. Europe or USA) and study protocol 12. Previous clinical trials performed in the UK have incorporated dose adjustments from a standard dose of 1.5 mg m^−2^ to 25 μg kg^−1^ from birth to 6 months of age and 1.0 mg m^−2^ from 6 months to 1 year or less than 10 kg body weight 13. Such age‐ or weight‐based cut‐off points can lead to instances where, once a threshold age or weight for a certain dose is reached, a patient will potentially receive a markedly increased dose following a relatively minor increase in body weight and/or age. Dosing recommendations for Act D have been revised on various occasions following concerns of under‐dosing or toxicity. However, no real scientific rationale currently exists for deciding the optimal dose of Act D in very young patients, with dose modifications based on caution and assumptions rather than a sound understanding of the disposition of Act D in these patients. Given the relative lack of clinical pharmacokinetic data available to guide dosing, the use of modelling and simulation methodologies offers an opportunity to predict the PK effects of various Act D doses and dosing regimens in different paediatric age ranges.

Physiologically based pharmacokinetic (PBPK) modelling and simulation is a method of PK analysis that can be used to predict the concentration–time course of a drug and its associated PK parameters using physiochemical and *in vitro* drug information as the basis for the model. In such a multi‐compartmental approach, major organs and tissues are represented by individual compartments which are arranged anatomically. In addition, nested models can be incorporated that describe the processes occurring in a particular organ. Nested models of the liver 14 and the kidney 15 may incorporate the metabolic processes of transporters, CYP enzymes and UGT enzymes, as well as the filtration and permeation of solutes into urine or bile. Commercially available PBPK software programs include virtual patient populations, which can be used to predict the impact of changing anatomy and physiology with age, in the case of paediatrics 16, 17, or pathophysiology of specific organs, in the case of hepatic or renal impairment 18. The use of PBPK modelling in the prediction of anticancer drug PKs in children has been described previously for methotrexate and etoposide 19, 20.

The aim of the current study was to develop a mechanistically based PBPK model for Act D in paediatric patients, with a view to facilitating the rational prediction of drug exposure in age ranges for which there are currently limited or no data to inform future dosing strategies.

## Methods

### Reagents

4‐methoxyphenol, *m*‐Cresol, 1‐naphthol, thymol, diphenyl ether, DMSO, Rosswell Park Memorial Institute (RPMI) 1640 medium with l‐glutamine, zinc sulphate and Act D were purchased from Sigma‐Aldrich (Poole, UK). Sodium nitrate was purchased from ReAgent (Cheshire, UK). Acetonitrile was provided by Fischer Scientific (Loughborough, UK). Foetal bovine serum and phenol red free Dulbecco's modified eagle medium (DMEM) were purchased from Gibco (Paisley, Scotland).

Drug/molecular target names used here are in accordance with the BJP's Guide to Receptor and Channels 21.

### Log *P*

The method for the generation of Log *P* values by high performance liquid chromatography (HPLC) was adapted from that described by Zhao *et al.* 22. Briefly, Log *P* correlates with the retention time of a compound by a solid phase C_18_ column. More lipophilic compounds partition more strongly onto the column and so are retained for a greater length of time than less lipophilic ones. By using compounds with known Log *P* values, a standard curve can be determined and the Log *P* of a compound of interest obtained by interpolation. A Waters HPLC system, linked to an Agilent fluorescence detector, was used along with a Luna 5 μ C_18_ 50 × 2.0 mm HPLC column preceded by a guard column (Phenomenex, Macclesfield, UK). Compounds with published Log *P* values (phenol, dexamethasone, chloramphenicol and verapamil) and sodium nitrate (a compound with a retention time of zero) were included as standards in the assay. Solutions of each of the standards (10 mM) were prepared in dimethyl sulfoxide (DMSO) and a 1 mg ml^−1^ solution of Act D was prepared in methanol. The sample holder for the HPLC was set at 25 °C in order to prevent freezing of the DMSO during the run. The mobile phase consisted of deionized water and acetonitrile with a constant gradient of 40–60%, respectively.

Prior to each run the column was equilibrated to the running conditions for 10 minutes, with the flow rate set to 0.5 ml min^−1^. A 20‐μl aliquot of each sample was injected into the column and its retention time measured relative to that of the sodium nitrate peak. Peaks were analysed using Empower software (Waters, Wilmslow, UK).

### Transporter kinetics

Previous work on the affinity of Act D for drug transporters was used to guide the *in vitro* assessment of transporter kinetics 23. In addition, the ability of the generated model to be extrapolated to other populations was assessed by attempting to predict Act D PK in adults, a population in which limited published data exist.

Polarized Madin‐Darby canine kidney (MDCKII) cell line wild‐type and cells transfected with *ABCB1* (MDR1) were obtained from Dr. A.H. Schinkel (Amsterdam, the Netherlands). Cells were cultured in RPMI 1640 medium supplemented with l‐glutamine and 10% foetal bovine serum, grown at 37 °C, 5% CO_2_ in a humidified incubator and were routinely screened for mycoplasma.

For drug transport experiments, 12‐mm Corning Transwell polycarbonate membrane cell culture inserts with 0.4‐μm pores were used (Sigma Aldrich, UK). Cells (passage 8–12) were seeded at 1.4 × 10^4^ cells ml^−1^ in growth medium and grown on filter for 5 days before the experiment was performed, with medium being changed every 24 hours. Fresh solutions of Act D were made at 0.1, 1, 10 and 100 μM in phenol red‐free Dulbecco's modified Eagle's medium (DMEM). Growth medium was aspirated from both the apical and basal compartments, and the cells were washed twice with pre‐warmed phosphate‐buffered saline adjusted to pH 7.4. Act D solutions (1.55 ml) were added to the basal chamber of their respective wells and phenol red‐free DMEM (0.5 ml) was added to the apical side of the monolayers. A 50‐μl sample from the initial Act D donor solutions was added to a 96‐well plate to be analysed alongside the post‐assay donor and receiver samples. Lucifer yellow (60 μM) was added as a paracellular control to test for the formation of a fully confluent monolayer. A limit of 2% Lucifer yellow movement was set as the cutoff with the results for any wells exceeding that threshold being rejected.

After 1 h, a 50‐μl aliquot of the experimental medium was taken from both the apical and basal chambers of each well and transferred to a 96‐well plate for dilution (1:2 with phenol red‐free DMEM) and quantification of Act D concentrations by liquid chromatography–mass spectrometry (LC–MS). An additional 50‐μl sample was taken and added to a 384‐well black plate for analysis of Lucifer yellow permeation. Analysis was carried out using a Luna 3 μ C8 (2) Mercury 20 × 4 mm column and a mobile phase consisting of: A acetate buffer (pH 4) and B methanol. After 10 min of conditioning at 35% A and 65% B, injections of 50 μl were made for each sample using gradient conditions as previously described 24.

The rate of efflux (μM min^−1^/10^6^ cells) due to MDR1 was determined by subtracting the rate of flux seen across the MDCKII‐WT cells from the flux across the MDCKII‐MDR1 cells. Cell counts were determined by re‐suspending the cells using trypsin and counting using a haemocytometer.

### Blood:plasma partitioning

An extraction solution of 1:1 deionized water and acetonitrile with 0.1% zinc sulphate was prepared on the day of the experiment and Act D solutions of 4, 20 and 100 μg ml^−1^ were prepared in saline. All experiments were performed in triplicate. An aliquot of 180 μl whole blood from a healthy donor was added to 20 μL of each Act D solution (final concentrations 0.4, 2 and 10 μg ml^−1^) in Eppendorf tubes, followed by gentle rotation for 1 min before transfer to an incubator (Infors HT Multitron Standard, Infors AG, Basel) at 37 °C for 1 h and 40 μl of plasma was transferred to a new Eppendorf and mixed with 40 μl of fresh blood.

For the blood samples, 40 μl of blood was transferred to a new Eppendorf and mixed with 40 μl blank plasma. All samples were then mixed with 160 μl of the extraction solution and frozen at −80 °C for 1 h. Samples were then centrifuged at 2000 g and 4 °C for 1 h and 100 μl of supernatant from each sample was transferred to HPLC vials and stored at −20 °C overnight. LC–MS analysis was performed as described above for the analysis of transporter kinetic samples. The blood‐to‐plasma ratio was calculated from the following equation (where *Kb*/*p* is the whole blood‐to‐plasma partition coefficient, *Ke*/*p* is the red blood cell‐to‐plasma partition and *H* is the haematocrit):

(1) $$\text{Blood}‐\mathrm{to}‐\text{plasma}\;\left(\mathrm{Kb}/p\right)=\left(\mathrm{Ke}/p\times H\right)+\left(1-H\right)$$

### Selection of clinical data

Ethical approval for the collection and use of patient data was obtained as part of the clinical trials involved, as previously described 6, 8, 10. Data generated from studies conducted in the UK were selected for the development of this model due to the quantity of information available for each individual subject. The data, previously published by Hill *et al*. 8, were initially separated into age groups of 1–<6, 6–<10 and 10–20 years of age. Data were then further categorized into dose bands, as different dosing regimens had been used. The banding of the clinical data is detailed in Table 1. Data from one of the four dose bands in the 10–20‐year‐old patient age range were used for model building, as they were the least likely to be affected by age‐related changes, while the remaining three dose bands were used for the model development set. The data for patients in the 1–<6 and 6–<10 year age bands were utilized for comparison with the simulated data obtained from the model. PK data for 30 adult patients were provided by St Bart's Hospital, London, for further assessment of the model 6.

**Table 1:** Summary of clinical cohorts used for model development; data obtained from 8

**Age range (years)**	**Dose (** **mg m** ^**−2**^ **)**	**n**	**Weight (kg) mean ± SD**	**BSA (** **m** ^**2**^ **) mean ± SD**	**% female**
**Development set**					
**10–20**	0.72	5	51.5 ± 14.1	1.5 ± 0.27	40
	1.05	4	75.3 ± 8.0	1.9 ± 0.11	25
	1.28	8	49.9 ± 7.9	1.5 ± 0.15	50
	1.5	6	45.1 ± 10.1	1.4 ± 0.19	16.7
**Test set**					
**6–<10**	0.77	5	31.9 ± 6.6	1.1 ± 0.15	40
	1.5	6	29.4 ± 3.3	1.0 ± 0.09	33
					
**1–<6**	0.6	11	10.4 ± 1.0	0.5 ± 0.03	55
	0.76	7	16.7 ± 3.3	0.7 ± 0.09	43
	1.01	16	13.6 ± 2.3	0.6 ± 0.07	69
	1.12	8	17.2 ± 4.6	0.7 ± 0.14	27
	1.48	14	18.0 ± 3.8	0.7 ± 0.11	50
	1.55	11	15.8 ± 3.6	0.7 ± 0.10	55
**16 to 53**	1	21		2.0 ± 0.16	
	0.8	9		2.0 ± 0.14	

### Modelling and simulation

#### Model development and initial simulations

Modelling and simulation were performed using Simcyp version 14 release 1 (Simcyp Ltd, a Certara company, Sheffield, UK). The compound file for Act D was compiled using data generated in the methods described above and with additional inputs from web databases and literature sources as shown in Table 2. The Simcyp software provides a framework for assessing the PK of a drug by defining certain characteristics within a compound file. Using this compound file, pharmacokinetic data can then be simulated in individuals of a virtual population of interest, allowing for the impact of inter‐individual differences to be assessed 17, 18.

**Table 2:** Summary of parameters utilized for the PBPK Act D model development

**Parameter**	**Value**	**Reference**
**Molecular weight**	1255.42 (g mol^−1^)	http://www.drugbank.ca/drugs/DB00970
**Log *P***	4.77	Current study
**Compound type**	Neutral	Determined from chemical structure
**Blood/plasma**	0.62	Current study
**Fraction unbound**	0.95	http://www.drugbank.ca/drugs/DB00970
**Distribution model**	Method 2	Rodgers and Rowland 25
**Kp scalar**	0.06	Determined by fitting using Lutz *et al.* 28
**Muscle distribution**	5.812	Modified from values in Lutz *et al.* 28
**Adipose distribution**	27.945	Modified from values in Lutz *et al.* 28
**CL**_**Renal**_	1.8 (l h^−1^)*	Tattersall *et al.* 5
**CL**_**Biliary**_	0.107 (μl min^−1^/10^6^ hepatocytes)*	Tattersall *et al.* 5
**CL**_**Additional**_	3.31 (l h^−1^)*	Fitted using parameter estimation – Nelder–Mead method.

* For these clearance values a CV of 60% was used.

For the simulation of clinical trial data, information was taken from previous work by Hill *et al*., who studied 117 patients administered Act D intravenously 8. The population was split into three age categories: 1–<6 (*n* = 67), 6–<10 (*n* = 11) and 10–20 years (*n* = 39). These age bins were further subdivided based on the dose of Act D received (Table 1). Simulations were run over 26 h following an IV bolus dose (relative to body surface area) administered over 3 min. A learn‐and‐confirm approach to model development was implemented, with initial simulations performed in a subset of the 10–20‐year‐old age range receiving the 1.24 mg kg^−1^ dose, using 10 trials of 10 virtual individuals, with half of the virtual population as females. These initial simulations were performed using the data shown in Table 2 with no fitting of parameters. Subsequent changes to parameters made during the development of the model are described in the results section.

In order to replicate the volume of distribution seen clinically, the tissue partition coefficients were set based on information from dogs 28. Once these values were incorporated, the muscle and adipose values were adjusted in order to fit the observed steady‐state volume of distribution from the prior clinical study 8. Running simulations with these partition coefficients yielded an initial systemic concentration comparable to that observed clinically.

As 50% of the accounted clearance of Act D is observed in faeces, two alternative methods for simulating the active biliary excretion of Act D were evaluated in the model development stage:

1. The kinetic data for *ABCB1* (MDR1) generated as part of this study was incorporated into the simulator for biliary transport of drug.
2. The biliary and renal clearance values were taken from a paper by Tattersall *et al.* 5. These data were incorporated into the PBPK model and used to generate *in vitro* data using the retrograde model. An additional clearance value was estimated using the parameter estimation function (Nelder–Mead method) within Simcyp to account for clearance of the drug that was not recovered.

#### Final simulations

Further evaluation of the final model was undertaken using adult PK data 6 made available from St Bart's Hospital (London, UK). Ten trials of 10 virtual subjects each were simulated in the default Caucasian healthy volunteer population, age range 21–64 years, with a proportion of females of 0.48. Paediatric simulations were performed in the 1–<6 and 6–<10 year populations from the study by Hill *et al.*, with age bin data further subdivided based on dose. Simulations were performed for all age bands and doses described in Table 1. The proportion of females was set as shown in Table 1 and a total of 100 subjects were simulated for each trial.

Simulations were then run to prospectively predict the Act D plasma PK profile in very young children. Simulations of 100 subjects given doses of 1.25 mg m^−2^ were run for three age groups: 0–<3 months, 3–<6 months and 6–12 months.

#### Final model evaluation

The Simcyp simulations output the mean and the 5th and 95th percentile for the concentration−time profile as well as the calculated mean, median and geometric mean of the simulated population. A visual predictive check of the simulated against observed data was performed on the final simulated results, including the number of data points within the 5th and 95th percentiles. In addition, the mean fold‐prediction of AUC against observed data was determined; a two‐fold under‐ or over‐prediction was considered a reasonable prediction and within 1.5‐fold a good prediction. The prediction of model variability was also assessed in a similar way by comparing coefficients of variation (CV).

## Results

### Log *P*

Using the HPLC method described, retention times were determined for all standards and Act D and *k* values were calculated using the following equation (where *tr* is the retention time of the compound of interest and *t*0 is the retention time of sodium nitrate):

(2) $$k=\frac{\mathrm{tr}-t0}{t0}$$

**Figure 1:**
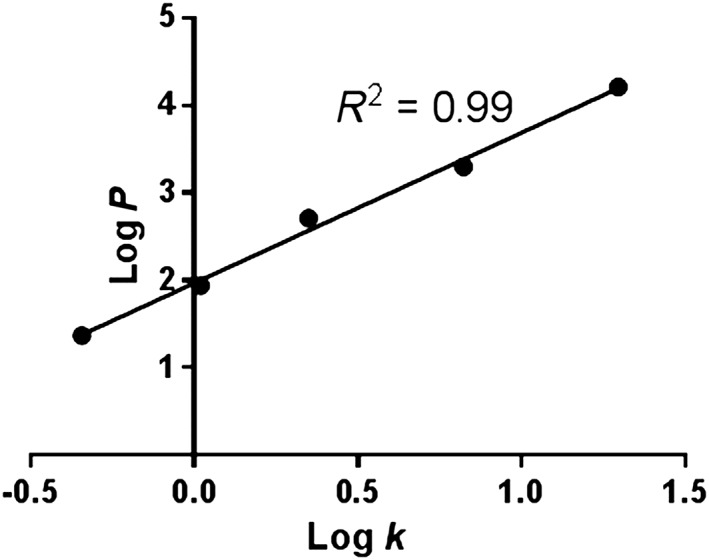
Standard curve of compounds with known Log *P* values. From left to right, the compounds are: 4‐methoxyphenol, m‐cresol, 1‐naphthol, thymol, diphenyl ether

Table 3 provides a summary of the results obtained. As shown in Figure 1, a correlation was determined between Log *k* and the known Log *P* values for the standards, and the Log *P* of Act D was extrapolated from the standard curve obtained. The Log *P* values for the standard compounds when determined by interpolation of the standard curve were all within 30% of their published values. The Log *P* of Act D was calculated as 4.47 (Table 2).

**Table 3:** Retention times of standard compounds and Act D and values used to determine the Log *P* of Act D

**Compound**	**Retention time (min)**	***k***	**Log *k***	**Experimental Log *P***	**Literature Log *P***	**Reference**
**Phenol**	0.81	0.75	−0.13	1.49	1.47	(Hansch *et al.*, 26)
**Dexamethasone**	0.97	1.10	0.04	1.80	1.83	(Lombardo *et al.* 27)
**Chloramphenicol**	0.77	0.66	−0.18	1.38	1.14	(Lombardo *et al.* 27)
**Verapamil**	4.19	80.2	0.90	3.45	3.79	(Hansch *et al.* 26)
**Act D**	13.94	28.72	1.46	4.47	—	

### Transporter kinetics

**Figure 2:**
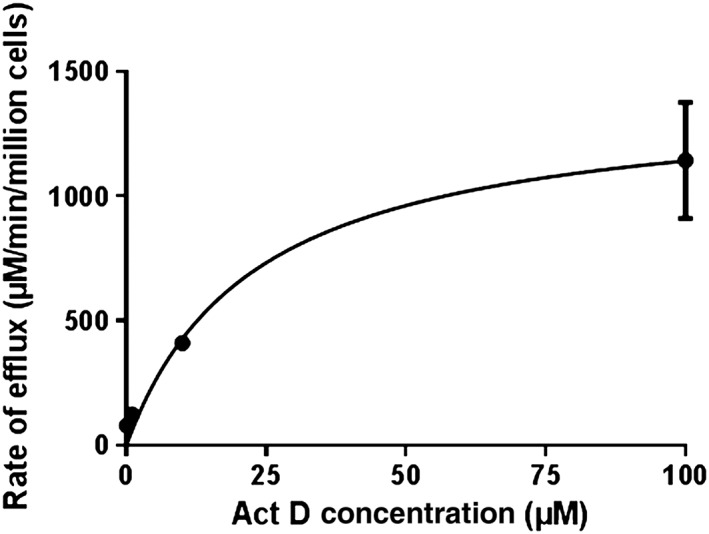
Graph showing the rate of movement of Act D across the MDCKII‐MDR1 monolayer at various concentrations

The Transwell™ assay to characterize the enzyme kinetics of transport of Act D by *ABCB1* (MDR1) determined the maximum rate of flux (*J*_max_) to be 1142 μM min^−1^/10^6^ cells, with a Michaelis–Menten constant (*K*_m_) of 20 μM. Figure 2 shows the rate of efflux *vs.* Act D concentration curve which appears to have reached a plateau by 100 μM.

### Blood‐to‐plasma partitioning

Concentrations of Act D in blood cells compared to concentrations in plasma were determined after spiking whole blood with drug concentrations ranging from 0.2 to 10 μg ml^−1^. Blood‐to‐plasma ratios between 0.59 and 0.65 were observed, with a mean blood‐to‐plasma ratio of 0.62 determined (95% confidence interval 0.03). This ratio was not dependent on Act D concentration.

### Simulations

Drug data for the compound file were taken from sources online and from the experiments detailed in this paper. Initial simulations were performed with the compound file data, including ABCB1 (MDR1) as a canalicular transporter in the liver, with no fitting. However, this resulted in an over‐prediction of the biliary elimination and a subsequent AUC that was lower than that derived from the clinical data.

**Figure 3:**
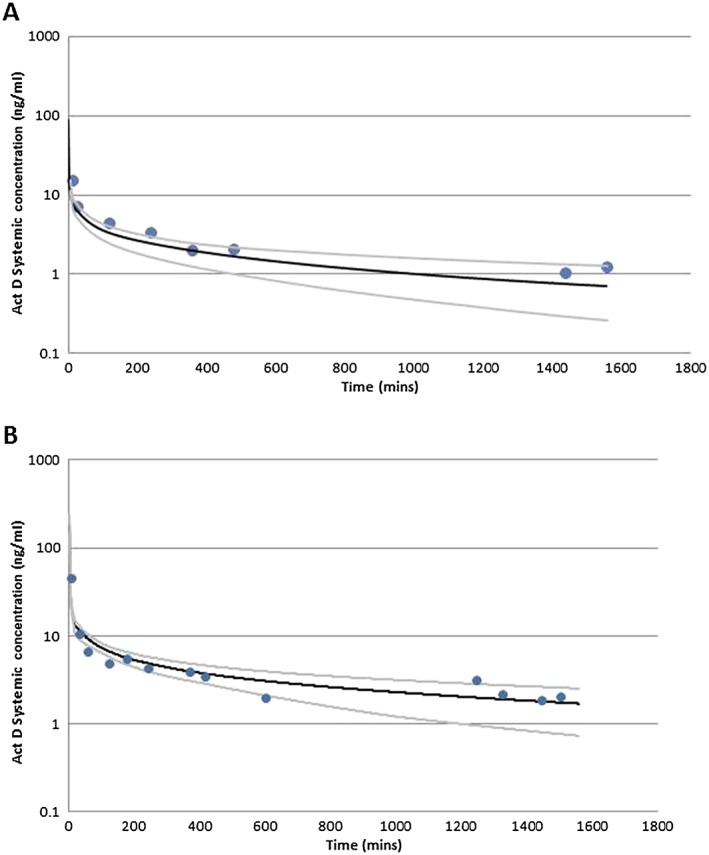
Visual predictive check showing the systemic concentration of Act D *vs.* time for clinical patients (circles) and mean (black line), 5th and 95th percentile (grey lines) of the simulated patient systemic Act D concentration. (A) Simulated and mean study patient Act D systemic concentration over time, 10–20 year old administered 1.29 mg m^−2^; (B) Simulated and mean study patient Act D systemic concentration over time, 1–6 year old administered 0.6 mg m^−2^

**Figure 4:**
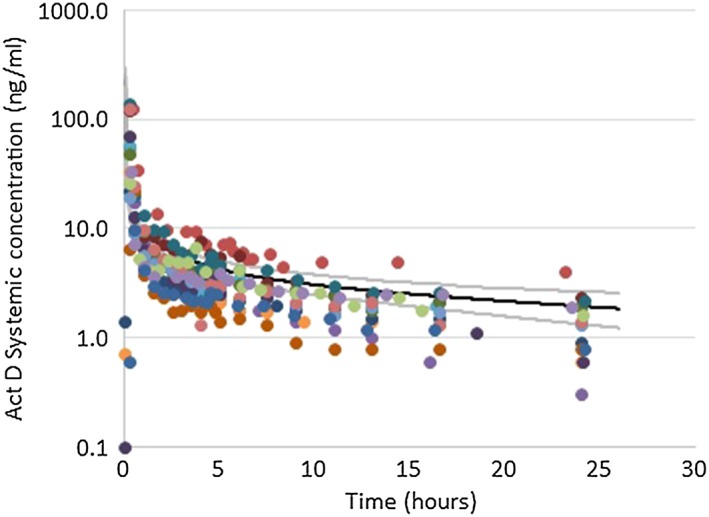
Systemic concentration of Act D *vs.* time for 21 adult clinical patients (circles) and mean (black line), 5th and 95th percentile (grey lines) of the simulated patient systemic Act D concentration over time, 16–53 year old administered 1 mg m^−2^

The final drug model incorporated clinical renal and biliary clearance values and an additional systemic clearance value to account for the differences observed in cumulative renal/biliary clearance and total observed systemic clearance (see Table 2 for values). Simulations using these clearance values were superimposed on the observed data (Figure 3A). Comparison of AUC_0‐26h_ in observed and simulated patients within the 10–20‐year‐old group showed all mean predictions to be within two‐fold of the mean observed values. The fold variability compared to observed values ranged from 1.2‐fold to 1.6‐fold, depending on dose group (Figure 3B). The model prediction was compared to data obtained from an adult PK study dataset 6. The visual predictive check (Figure 4) showed that the simulated mean concentration–time profile fell within the observed results; however, the simulation failed to capture the full variability of the observed data (observed AUC_0‐26h_ SD: 595.33 ng h ml^−1^, simulated AUC_0‐26h_ SD: 9.04 ng h ml^−1^).

**Figure 5:**
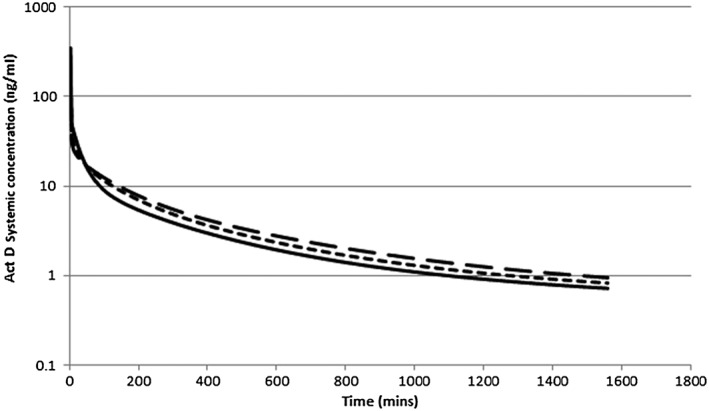
Systemic concentration of Act D *vs.* time for 100 simulated patients less than one year of age given 1.25 mg m^−2^ IV mean, 5th and 95th (0–<3 months, 3–<6 months, 6–12 months)

For the younger age ranges (example profile, Figure 3B), all AUC predictions were within two‐fold of the observed values, with simulated data from six of the eight age/dose ranges falling within 15% of the observed data (Table 4). The level of variability as compared to the observed values ranged from 1‐ to 1.8‐fold. Simulations for very young children (0–<3, 3–<6 and 6–12 months old) showed comparable PK profile shape to the older children, with mean AUC_0‐26h_ values of 104.07, 109.99 and 115.42 ng h ml^−1^ and clearance values of 3.5, 3.6 and 3.8 l h^−1^ determined, respectively (Figure 5).

**Table 4:** Comparison of AUC_0‐26h_ observed clinically and simulated for patients of different ages and doses

**Age group (years)**	**Dose (** **mg m** ^**−2**^ **)**	**Mean simulated AUC (ng h ml^−1^) ± SD**	**Mean observed AUC (ng h ml^−1^) ± SD**	**Simulated/Observed ± SD**
**10–20** *	0.74	57 ± 11	45 ± 8	1.27 ± 0.33
	1.04	77 ± 16	50 ± 12	1.54 ± 0.49
	1.48	118 ± 25	120 ± 44	0.98 ± 0.42
**6–<10**	0.77	67 ± 11	80 ± 28	0.84 ± 0.32
	1.50	130 ± 21	130 ± 12	1.00 ± 0.19
**1–<6**	0.60	57 ± 12	82 ± 18	0.70 ± 0.21
	0.76	72 ± 15	63 ± 8	0.14 ± 0.28
	1.01	94 ± 20	71 ± 33	1.32 ± 0.68
	1.12	106 ± 23	82 ± 16	1.29 ± 0.38
	1.48	136 ± 30	111 ± 16	1.22 ± 0.32
	1.55	146 ± 33	128 ± 51	1.14 ± 0.52

* The 10–20‐year‐old group was used to determine the best level at which to set the variability.

## Discussion

The current study describes the development of a PBPK model for the prediction of Act D exposure in children using a combination of experimentally determined and literature‐derived system parameters. The model described shows an ability to predict the systemic exposure of Act D across a range of ages and doses, with all simulated AUC values falling within two‐fold and in six out of eight cases within 15% of those observed in clinical pharmacokinetic studies. The model has the potential to be used for the prediction of Act D exposure in very young cancer patients, including neonates and infants less than one year old, and may help guide future dosing in a clinical setting. Ultimately, however, independent data from neonates and infants will be required for full validation of this model, especially in children less than one year of age in whom virtually no Act D PK data exist. Indeed, this reflects a wider problem in cancer therapy, with PK data in neonates and very young infants being extremely difficult to obtain but urgently needed if PBPK models are to be used with confidence to guide dosing in this most vulnerable population. This holds true for virtually all anticancer drugs used in neonates and infants. With the recent revocation of paediatric investigation plan waivers for a number of drug classes, PBPK modelling has the potential to be of use, informing initial dose selection and trial design for these drugs 29.

Although this PBPK model for Act D accurately predicts the mean values of the adult data profiles, it failed to capture the full variability observed in the adult PK dataset. One reason for this may be that while the CL_Renal_ was determined mechanistically as described in the methods section, the variability of biliary and additional clearances in the model within Simcyp were determined by the %CV entered into the compound file. This was set at 60% based on the variability seen in the development population of 10–20 year olds given the relatively small set of data used for development. This may under‐estimate the true variability of Act D PK in the Caucasian adult population. Another factor is the impact of co‐administered drugs such as vincristine. As 90% of patients were receiving both Act D and vincristine, it was not possible to determine any impact of co‐administration.

In addition, healthy virtual adult and also paediatric populations were used in this study. These populations do not necessarily reflect physiological changes occurring in the setting of cancer which can affect drug disposition. Such changes may include increased alpha 1 acid glycoprotein and decreased albumin concentrations 30 as well as increases in inflammatory markers which may suppress enzyme and transporter function 31, 32, decreases in GFR after nephrotoxic drug treatments, and body mass alterations due to cachexia. These changes could have a direct impact on the PK of any drugs given to patients with existing conditions.

These same physiological changes occurring in the paediatric cancer population are complicated by the known effects of age and development in all paediatric populations on factors such as renal function, plasma protein concentrations, enzyme and transporter expression, body surface area, and organ size and blood flow 17. Analysis of demographic and laboratory data from childhood cancer patients is currently being undertaken with a view towards characterizing the differences between ontogeny functions in paediatric cancer patients as compared with healthy children and building a virtual paediatric cancer population for use in PBPK modelling and simulation in paediatric cancer 33.

In addition, it is also feasible that Act D disposition in humans has not been fully characterized and therefore unknown mechanisms of clearance or transport may exist that could not be incorporated into the model. It has been suggested that Act D binds to DNA at specific regions 1, hence it is possible that irreversible binding of Act D to DNA may reflect a clearance pathway that has not yet been elucidated. Further research into the clearance of Act D and how it changes with age may help increase confidence in these predictive simulations.

Finally, a greater understanding of the specific transporters involved in the clearance of Act D is required, as well as an improved knowledge of the absolute expression and activity of those transporters in the relevant tissues and their ontogeny. The availability of these data would clearly help with the development of future PBPK models in paediatric oncology.

The ability of the model described to predict Act D AUC in a variety of paediatric age ranges suggests that it is possible to develop PBPK models for anticancer drugs in populations where minimal datasets exist. As was the case in the current study, such approaches may require the generation of physiochemical and *in vitro* drug information not available through published literature and database searches. As more information on ontogeny of physiological processes becomes available, predictive PK models have the potential to improve chemotherapy dosing for the youngest patients who may be most at risk of adverse effects following treatment. PBPK also provides the scope to investigate specific tissue distribution of drugs as well as to incorporate new data both in the form of model specification or physiological data, without the need to perform further clinical trials.

## Competing Interests

All authors have completed the Unified Competing Interest form at http://www.icmje.org/coi_disclosure.pdf (available on request from the corresponding author) and declare no support from any organization for the submitted work. Dr Trevor Johnson and Dr Sibylle Neuhoff are employees of Simcyp Ltd (a Certara Company). Simcyp's research is funded by a consortium of pharmaceutical companies. The Simcyp Simulator is freely available, following completion of the training workshop, to approved members of academic institutions and other not‐for‐profit organizations for research or teaching purposes. The authors have no other relationships or activities that could appear to have influenced the submitted work.

This work was supported in part by the Medical Research Council, the NIHR Newcastle Biomedical Research Centre and Cancer Research UK.

## Contributors

CW carried out the experimental work outlined in the paper and development of the PBPK model. JB, TJ, SH, AB and GV provided input into the model development and assistance with manuscript preparation. EG and JG were responsible for the provision of clinical data from the adult clinical trial. All authors read and approved the final version of the manuscript.
